# Poster Session I - A40 SHORT-CHAIN FATTY ACIDS AS POTENTIAL MODULATORS OF TYPE III INTERFERON SIGNALING THROUGH EPIGENETIC REGULATION

**DOI:** 10.1093/jcag/gwaf042.040

**Published:** 2026-02-13

**Authors:** T Olumade, Y Fedorova, C Lantin, D Santer

**Affiliations:** Immunology, University of Manitoba Faculty of Health Sciences, Winnipeg, MB, Canada; Immunology, University of Manitoba Faculty of Health Sciences, Winnipeg, MB, Canada; Immunology, University of Manitoba Faculty of Health Sciences, Winnipeg, MB, Canada; Immunology, University of Manitoba Faculty of Health Sciences, Winnipeg, MB, Canada

## Abstract

**Background:**

Type III interferons (IFN-λs) are highly expressed in the gut, where they promote mucosal healing and dampen inflammation. IFN-λs regulate only on a subset of cells – such as gut epithelial cells and certain immune cells like macrophages – due to restricted expression of IFN-λR1. Prior studies have shown that short-chain fatty acids (SCFAs), one class of microbial-derived metabolites, act as histone deacetylase (HDAC) inhibitors, modulating gene expression to improve gut barrier integrity and supporting immune homeostasis. However, while both SCFAs and IFN-λs contribute to gut homeostasis, any potential synergistic effects are not well understood.

**Aims:**

This project initially aims to uncover which metabolites found in the gut regulate IFN-λ activity, and whether the effect is cell-type specific. We hypothesized that SCFAs would promote IFN-λ responsiveness of both immune cells and epithelial cells.

**Methods:**

Caco-2 cells and primary monocyte-derived macrophages were pre-treated for 2 hours with gut metabolites, including SCFAs (acetate, butyrate, propionate), bile acids, kynurenine, and succinate. The cells were then treated with or without IFN-λ3 (50ng/ml) for 24 hours (n = 3-4 independent experiments). IFN-stimulated genes (ISGs; *MX1* or *IFITI*) and *IFNLR1* expression were quantified by RT-qPCR. Surface IFN-λR1 and IL-10RB levels were also quantified by flow cytometry. To chemically mimic SCFA-induced HDAC inhibition, Caco-2 cells were pre-treated with MS-275 as described above. Cell viability was measured by PrestoBlue assay.

**Results:**

All SCFAs tested inhibited ISG induction by IFN-λ3 (p < 0.05) in Caco-2 cells, but not macrophages, with no changes in cell viability. Interestingly, basal levels of ISG expression instead were increased in macrophages with all SCFAs added alone. Other metabolites tested did not affect ISG induction at baseline or after IFN-λ3 addition at any concentration tested. No metabolites significantly altered *IFNLR1* mRNA expression. However, acetate upregulated, butyrate significantly downregulated, and propionate did not affect IFN-λR1 surface levels on Caco-2 cells. MS-275 treatment inhibited ISG induction by IFN-λ3 in Caco-2 cells, suggesting that SCFAs may inhibit IFN-λ3-mediated induction of ISGs through HDAC inhibition.

**Conclusions:**

Our findings demonstrate that SCFAs differentially modulate IFN-λ responses and surface IFN-λR1 levels depending on the cell type, possibly related to HDAC inhibition. Although the inhibition of ISG induction was contrary to our hypothesis, further work is ongoing to confirm these observations in primary gut epithelial cells. Nevertheless, this study adds to our fundamental knowledge of how IFN-λ biology could be regulated in the human gut.

**Funding Agencies:**

CAG, CCC, CIHRTRIANGLE, University of Manitoba

